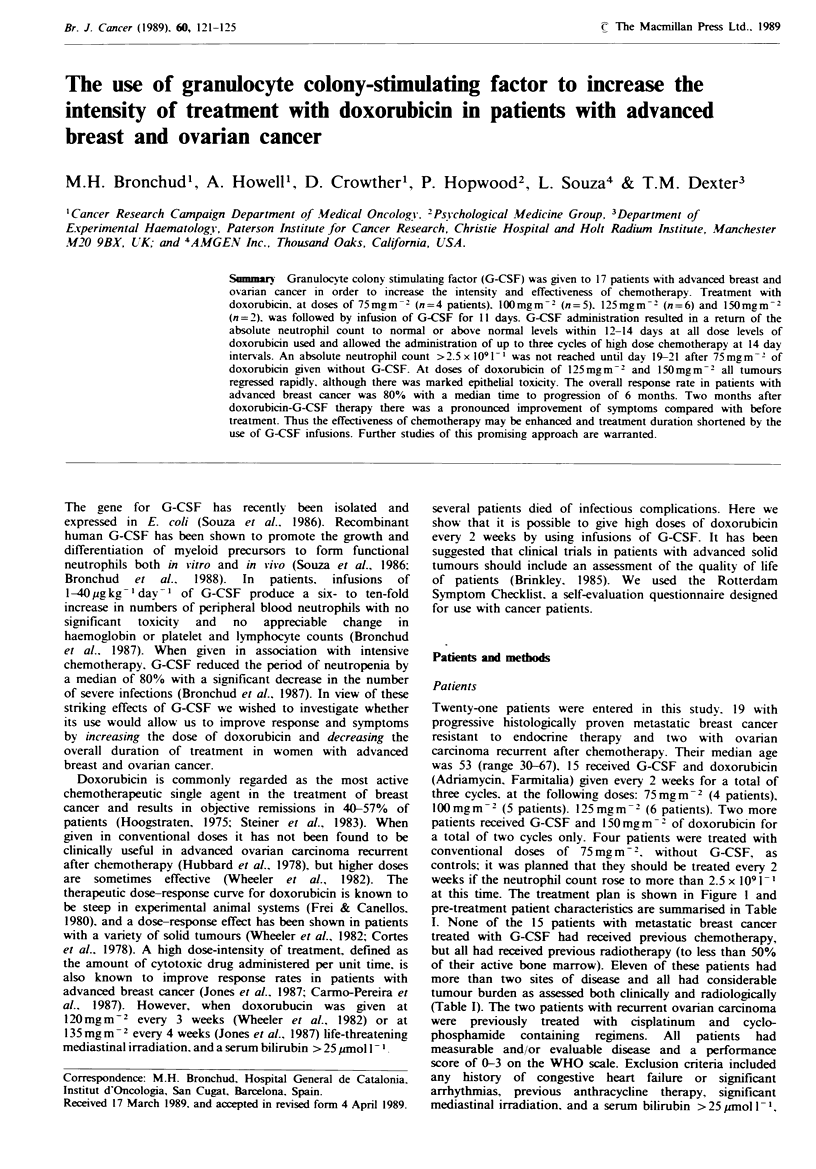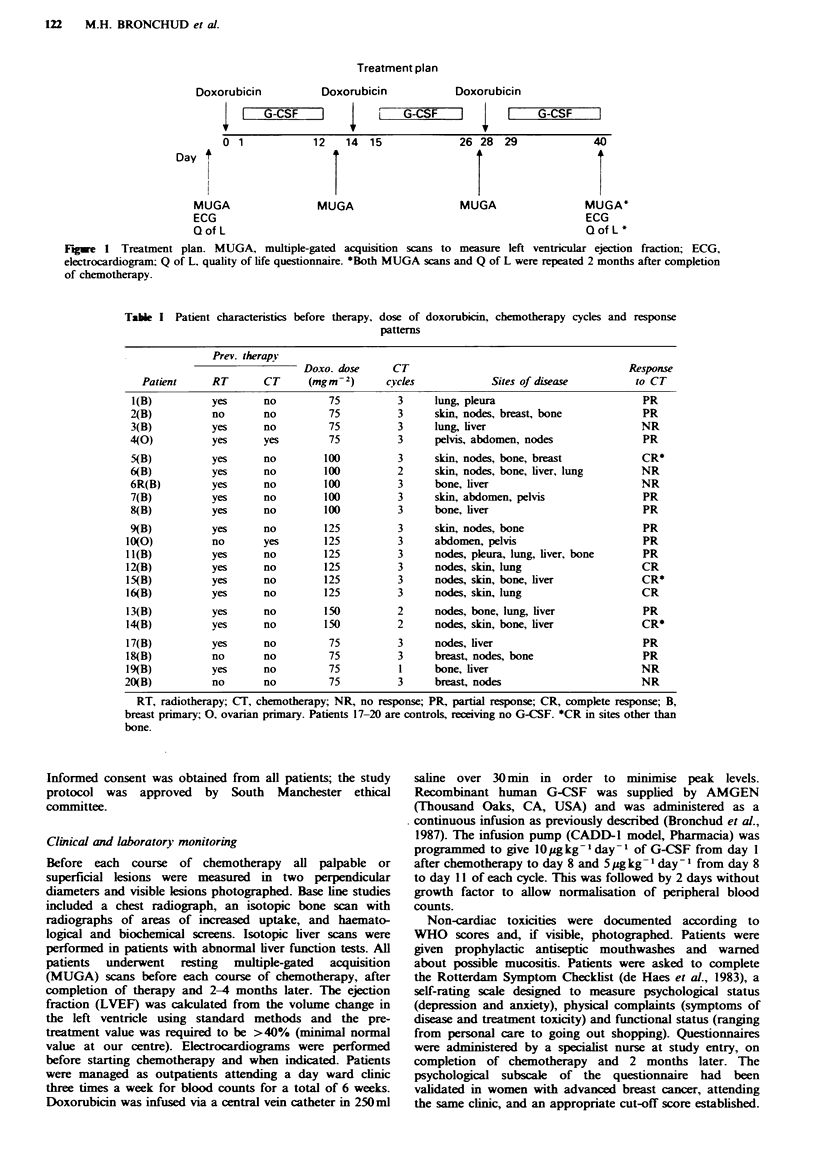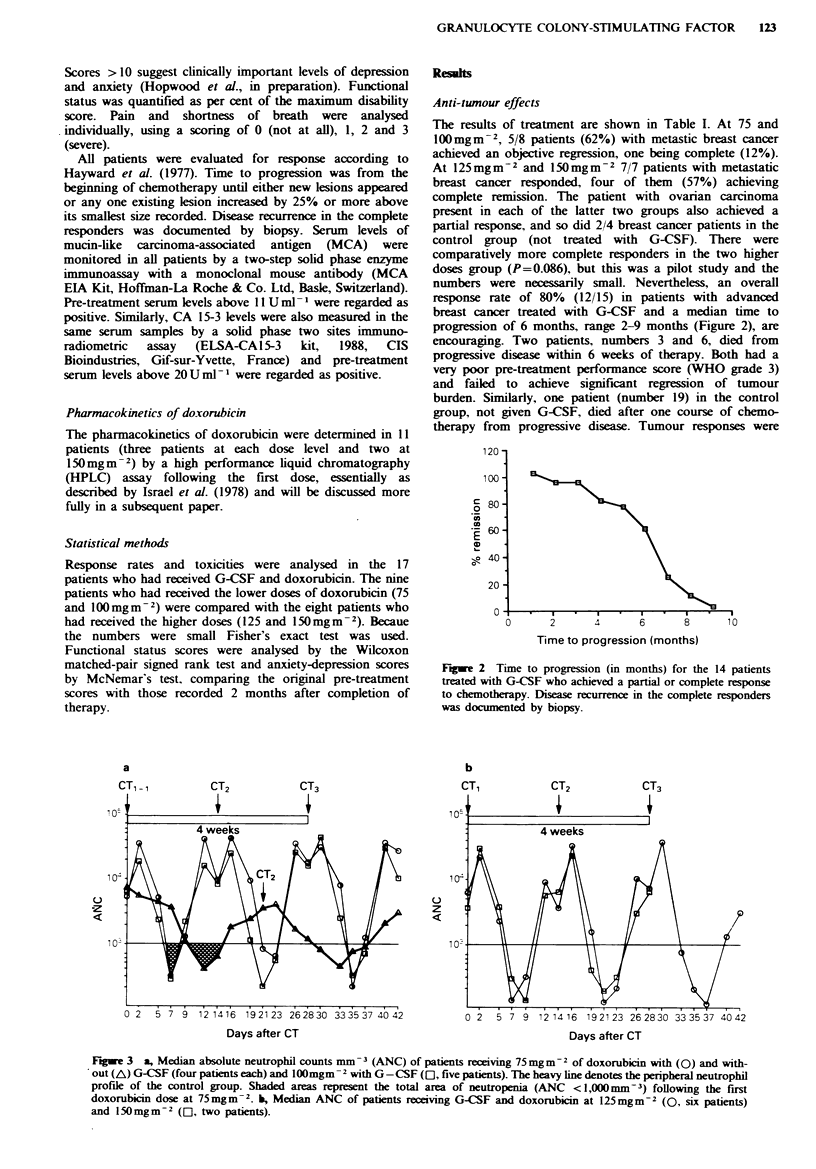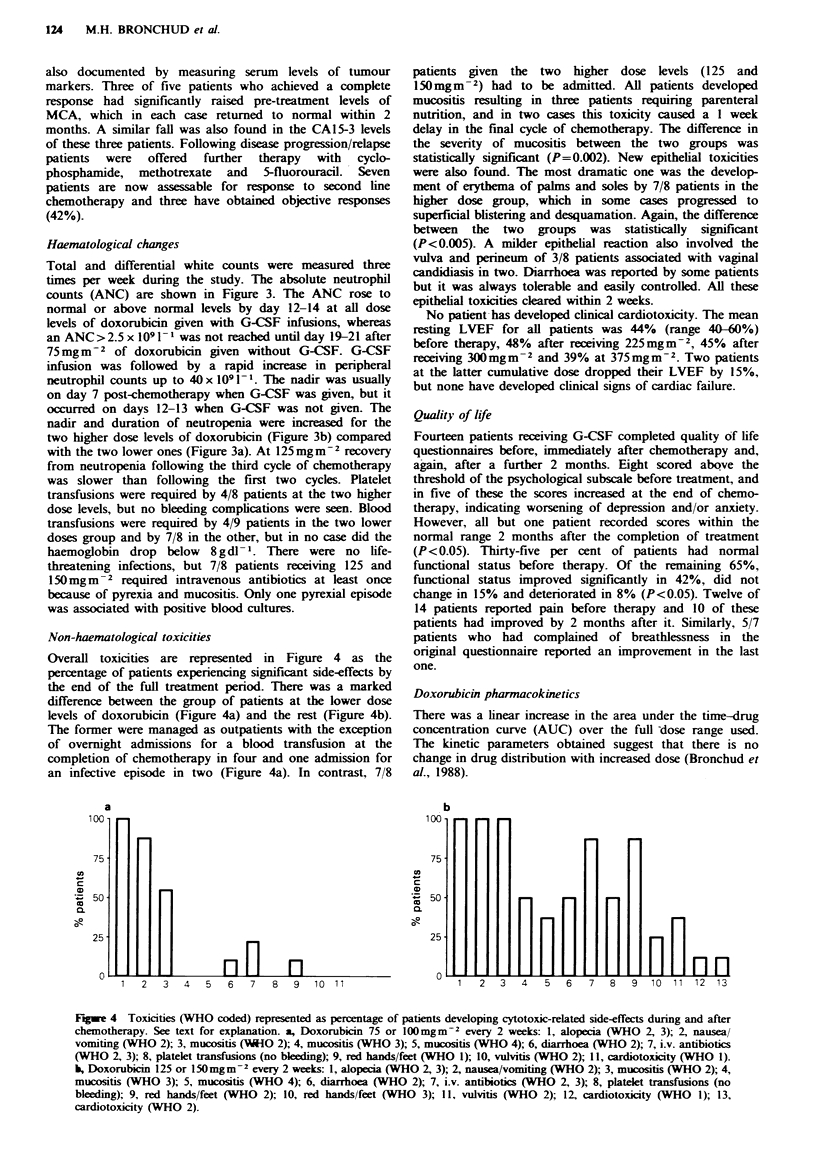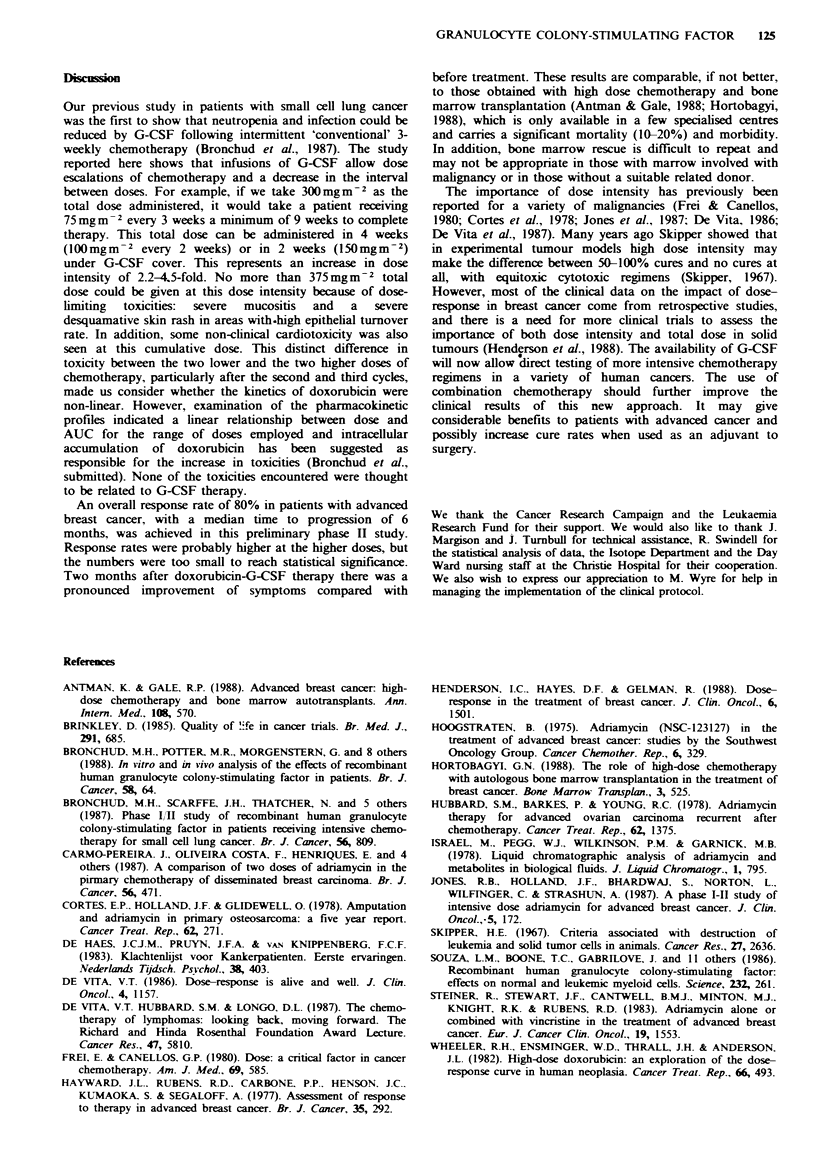# The use of granulocyte colony-stimulating factor to increase the intensity of treatment with doxorubicin in patients with advanced breast and ovarian cancer.

**DOI:** 10.1038/bjc.1989.234

**Published:** 1989-07

**Authors:** M. H. Bronchud, A. Howell, D. Crowther, P. Hopwood, L. Souza, T. M. Dexter

**Affiliations:** Cancer Research Campaign Department of Medical Oncology, Paterson Institute for Cancer Research, Christie Hospital and Holt Radium Institute, Manchester, UK.

## Abstract

Granulocyte colony stimulating factor (G-CSF) was given to 17 patients with advanced breast and ovarian cancer in order to increase the intensity and effectiveness of chemotherapy. Treatment with doxorubicin, at doses of 75 mg m-2 (n = 4 patients), 100 mg m-2 (n = 5), 125 mg m-2 (n = 6) and 150 mg m-2 (n = 2), was followed by infusion of G-CSF for 11 days. G-CSF administration resulted in a return of the absolute neutrophil count to normal or above normal levels within 12-14 days at all dose levels of doxorubicin used and allowed the administration of up to three cycles of high dose chemotherapy at 14 day intervals. An absolute neutrophil count greater than 2.5 x 10(9)l-1 was not reached until day 19-21 after 75 mg m-2 of doxorubicin given without G-CSF. At doses of doxorubicin of 125 mg m-2 and 150 mg m-2 all tumours regressed rapidly, although there was marked epithelial toxicity. The overall response rate in patients with advanced breast cancer was 80% with a median time to progression of 6 months. Two months after doxorubicin-G-CSF therapy there was a pronounced improvement of symptoms compared with before treatment. Thus the effectiveness of chemotherapy may be enhanced and treatment duration shortened by the use of G-CSF infusions. Further studies of this promising approach are warranted.


Br. J. Cancer (1989), 60, 121–125 © The Macmillan Press Ltd., 1989

### The use of granulocyte colony-stimulating factor to increase the intensity of treatment with doxorubicin in patients with advanced breast and ovarian cancer

M.H. Bronchud[1], A. Howell[1], D. Crowther[1], P. Hopwood[2], L. Souza[4] & T.M. Dexter[3]

[1]*Cancer Research Campaign Department of Medical Oncology*, [2]*Psychological Medicine Group*, [3]*Department of Experimental Haematology, Paterson Institute for Cancer Research, Christie Hospital and Holt Radium Institute, Manchester M20 9BX, UK; and* [4]*AMGEN Inc., Thousand Oaks, California, USA.*

**Summary** Granulocyte colony stimulating factor (G-CSF) was given to 17 patients with advanced breast and ovarian cancer in order to increase the intensity and effectiveness of chemotherapy. Treatment with doxorubicin, at doses of $75\,mg\,m^{-2}$ ($n=4$ patients), $100\,mg\,m^{-2}$ ($n=5$), $125\,mg\,m^{-2}$ ($n=6$) and $150\,mg\,m^{-2}$ ($n=2$), was followed by infusion of G-CSF for 11 days. G-CSF administration resulted in a return of the absolute neutrophil count to normal or above normal levels within 12–14 days at all dose levels of doxorubicin used and allowed the administration of up to three cycles of high dose chemotherapy at 14 day intervals. An absolute neutrophil count $>2.5\times10^9\,l^{-1}$ was not reached until day 19–21 after $75\,mg\,m^{-2}$ of doxorubicin given without G-CSF. At doses of doxorubicin of $125\,mg\,m^{-2}$ and $150\,mg\,m^{-2}$ all tumours regressed rapidly, although there was marked epithelial toxicity. The overall response rate in patients with advanced breast cancer was 80% with a median time to progression of 6 months. Two months after doxorubicin-G-CSF therapy there was a pronounced improvement of symptoms compared with before treatment. Thus the effectiveness of chemotherapy may be enhanced and treatment duration shortened by the use of G-CSF infusions. Further studies of this promising approach are warranted.

The gene for G-CSF has recently been isolated and expressed in *E. coli* (Souza *et al.*, 1986). Recombinant human G-CSF has been shown to promote the growth and differentiation of myeloid precursors to form functional neutrophils both *in vitro* and *in vivo* (Souza *et al.*, 1986; Bronchud *et al.*, 1988). In patients, infusions of $1–40\,\mu g\,kg^{-1}\,day^{-1}$ of G-CSF produce a six- to ten-fold increase in numbers of peripheral blood neutrophils with no significant toxicity and no appreciable change in haemoglobin or platelet and lymphocyte counts (Bronchud *et al.*, 1987). When given in association with intensive chemotherapy, G-CSF reduced the period of neutropenia by a median of 80% with a significant decrease in the number of severe infections (Bronchud *et al.*, 1987). In view of these striking effects of G-CSF we wished to investigate whether its use would allow us to improve response and symptoms by *increasing* the dose of doxorubicin and *decreasing* the overall duration of treatment in women with advanced breast and ovarian cancer.

Doxorubicin is commonly regarded as the most active chemotherapeutic single agent in the treatment of breast cancer and results in objective remissions in 40–57% of patients (Hoogstraten, 1975; Steiner *et al.*, 1983). When given in conventional doses it has not been found to be clinically useful in advanced ovarian carcinoma recurrent after chemotherapy (Hubbard *et al.*, 1978), but higher doses are sometimes effective (Wheeler *et al.*, 1982). The therapeutic dose–response curve for doxorubicin is known to be steep in experimental animal systems (Frei & Canellos, 1980), and a dose–response effect has been shown in patients with a variety of solid tumours (Wheeler *et al.*, 1982; Cortes *et al.*, 1978). A high dose-intensity of treatment, defined as the amount of cytotoxic drug administered per unit time, is also known to improve response rates in patients with advanced breast cancer (Jones *et al.*, 1987; Carmo-Pereira *et al.*, 1987). However, when doxorubucin was given at $120\,mg\,m^{-2}$ every 3 weeks (Wheeler *et al.*, 1982) or at $135\,mg\,m^{-2}$ every 4 weeks (Jones *et al.*, 1987) life-threatening mediastinal irradiation, and a serum bilirubin $>25\,\mu mol\,l^{-1}$.

Correspondence: M.H. Bronchud, Hospital General de Catalonia, Institut d'Oncologia, San Cugat, Barcelona, Spain.
Received 17 March 1989, and accepted in revised form 4 April 1989.

several patients died of infectious complications. Here we show that it is possible to give high doses of doxorubicin every 2 weeks by using infusions of G-CSF. It has been suggested that clinical trials in patients with advanced solid tumours should include an assessment of the quality of life of patients (Brinkley, 1985). We used the Rotterdam Symptom Checklist, a self-evaluation questionnaire designed for use with cancer patients.

### Patients and methods

### *Patients*

Twenty-one patients were entered in this study, 19 with progressive histologically proven metastatic breast cancer resistant to endocrine therapy and two with ovarian carcinoma recurrent after chemotherapy. Their median age was 53 (range 30–67), 15 received G-CSF and doxorubicin (Adriamycin, Farmitalia) given every 2 weeks for a total of three cycles, at the following doses: $75\,mg\,m^{-2}$ (4 patients), $100\,mg\,m^{-2}$ (5 patients), $125\,mg\,m^{-2}$ (6 patients). Two more patients received G-CSF and $150\,mg\,m^{-2}$ of doxorubicin for a total of two cycles only. Four patients were treated with conventional doses of $75\,mg\,m^{-2}$, without G-CSF, as controls; it was planned that they should be treated every 2 weeks if the neutrophil count rose to more than $2.5\times10^9\,l^{-1}$ at this time. The treatment plan is shown in Figure 1 and pre-treatment patient characteristics are summarised in Table I. None of the 15 patients with metastatic breast cancer treated with G-CSF had received previous chemotherapy, but all had received previous radiotherapy (to less than 50% of their active bone marrow). Eleven of these patients had more than two sites of disease and all had considerable tumour burden as assessed both clinically and radiologically (Table I). The two patients with recurrent ovarian carcinoma were previously treated with cisplatinum and cyclophosphamide containing regimens. All patients had measurable and/or evaluable disease and a performance score of 0–3 on the WHO scale. Exclusion criteria included any history of congestive heart failure or significant arrhythmias, previous anthracycline therapy, significant mediastinal irradiation, and a serum bilirubin $>25\,\mu mol\,l^{-1}$,

**Figure 1** Treatment plan. MUGA, multiple-gated acquisition scans to measure left ventricular ejection fraction; ECG, electrocardiogram; Q of L, quality of life questionnaire. *Both MUGA scans and Q of L were repeated 2 months after completion of chemotherapy.

**Table I** Patient characteristics before therapy, dose of doxorubicin, chemotherapy cycles and response patterns

| Patient | Prev. therapy RT | Prev. therapy CT | Doxo. dose ($mg\,m^{-2}$) | CT cycles | Sites of disease | Response to CT |
|---|---|---|---|---|---|---|
| 1(B) | yes | no | 75 | 3 | lung, pleura | PR |
| 2(B) | no | no | 75 | 3 | skin, nodes, breast, bone | PR |
| 3(B) | yes | no | 75 | 3 | lung, liver | NR |
| 4(O) | yes | yes | 75 | 3 | pelvis, abdomen, nodes | PR |
| 5(B) | yes | no | 100 | 3 | skin, nodes, bone, breast | CR* |
| 6(B) | yes | no | 100 | 2 | skin, nodes, bone, liver, lung | NR |
| 6R(B) | yes | no | 100 | 3 | bone, liver | NR |
| 7(B) | yes | no | 100 | 3 | skin, abdomen, pelvis | PR |
| 8(B) | yes | no | 100 | 3 | bone, liver | PR |
| 9(B) | yes | no | 125 | 3 | skin, nodes, bone | PR |
| 10(O) | no | yes | 125 | 3 | abdomen, pelvis | PR |
| 11(B) | yes | no | 125 | 3 | nodes, pleura, lung, liver, bone | PR |
| 12(B) | yes | no | 125 | 3 | nodes, skin, lung | CR |
| 15(B) | yes | no | 125 | 3 | nodes, skin, bone, liver | CR* |
| 16(B) | yes | no | 125 | 3 | nodes, skin, lung | CR |
| 13(B) | yes | no | 150 | 2 | nodes, bone, lung, liver | PR |
| 14(B) | yes | no | 150 | 2 | nodes, skin, bone, liver | CR* |
| 17(B) | yes | no | 75 | 3 | nodes, liver | PR |
| 18(B) | no | no | 75 | 3 | breast, nodes, bone | PR |
| 19(B) | yes | no | 75 | 1 | bone, liver | NR |
| 20(B) | no | no | 75 | 3 | breast, nodes | NR |

RT, radiotherapy; CT, chemotherapy; NR, no response; PR, partial response; CR, complete response; B, breast primary; O, ovarian primary. Patients 17–20 are controls, receiving no G-CSF. *CR in sites other than bone.

Informed consent was obtained from all patients; the study protocol was approved by South Manchester ethical committee.

### *Clinical and laboratory monitoring*

Before each course of chemotherapy all palpable or superficial lesions were measured in two perpendicular diameters and visible lesions photographed. Base line studies included a chest radiograph, an isotopic bone scan with radiographs of areas of increased uptake, and haematological and biochemical screens. Isotopic liver scans were performed in patients with abnormal liver function tests. All patients underwent resting multiple-gated acquisition (MUGA) scans before each course of chemotherapy, after completion of therapy and 2–4 months later. The ejection fraction (LVEF) was calculated from the volume change in the left ventricle using standard methods and the pre-treatment value was required to be >40% (minimal normal value at our centre). Electrocardiograms were performed before starting chemotherapy and when indicated. Patients were managed as outpatients attending a day ward clinic three times a week for blood counts for a total of 6 weeks. Doxorubicin was infused via a central vein catheter in 250 ml saline over 30 min in order to minimise peak levels. Recombinant human G-CSF was supplied by AMGEN (Thousand Oaks, CA, USA) and was administered as a continuous infusion as previously described (Bronchud *et al.*, 1987). The infusion pump (CADD-1 model, Pharmacia) was programmed to give $10\,\mu g\,kg^{-1}\,day^{-1}$ of G-CSF from day 1 after chemotherapy to day 8 and $5\,\mu g\,kg^{-1}\,day^{-1}$ from day 8 to day 11 of each cycle. This was followed by 2 days without growth factor to allow normalisation of peripheral blood counts.

Non-cardiac toxicities were documented according to WHO scores and, if visible, photographed. Patients were given prophylactic antiseptic mouthwashes and warned about possible mucositis. Patients were asked to complete the Rotterdam Symptom Checklist (de Haes *et al.*, 1983), a self-rating scale designed to measure psychological status (depression and anxiety), physical complaints (symptoms of disease and treatment toxicity) and functional status (ranging from personal care to going out shopping). Questionnaires were administered by a specialist nurse at study entry, on completion of chemotherapy and 2 months later. The psychological subscale of the questionnaire had been validated in women with advanced breast cancer, attending the same clinic, and an appropriate cut-off score established.

Scores >10 suggest clinically important levels of depression and anxiety (Hopwood *et al.*, in preparation). Functional status was quantified as per cent of the maximum disability score. Pain and shortness of breath were analysed individually, using a scoring of 0 (not at all), 1, 2 and 3 (severe).

All patients were evaluated for response according to Hayward *et al.* (1977). Time to progression was from the beginning of chemotherapy until either new lesions appeared or any one existing lesion increased by 25% or more above its smallest size recorded. Disease recurrence in the complete responders was documented by biopsy. Serum levels of mucin-like carcinoma-associated antigen (MCA) were monitored in all patients by a two-step solid phase enzyme immunoassay with a monoclonal mouse antibody (MCA EIA Kit, Hoffman-La Roche & Co. Ltd, Basle, Switzerland). Pre-treatment serum levels above 11 U ml$^{-1}$ were regarded as positive. Similarly, CA 15-3 levels were also measured in the same serum samples by a solid phase two sites immunoradiometric assay (ELSA-CA15-3 kit, 1988, CIS Bioindustries, Gif-sur-Yvette, France) and pre-treatment serum levels above 20 U ml$^{-1}$ were regarded as positive.

*Pharmacokinetics of doxorubicin*

The pharmacokinetics of doxorubicin were determined in 11 patients (three patients at each dose level and two at 150 mg m$^{-2}$) by a high performance liquid chromatography (HPLC) assay following the first dose, essentially as described by Israel *et al.* (1978) and will be discussed more fully in a subsequent paper.

*Statistical methods*

Response rates and toxicities were analysed in the 17 patients who had received G-CSF and doxorubicin. The nine patients who had received the lower doses of doxorubicin (75 and 100 mg m$^{-2}$) were compared with the eight patients who had received the higher doses (125 and 150 mg m$^{-2}$). Becaue the numbers were small Fisher's exact test was used. Functional status scores were analysed by the Wilcoxon matched-pair signed rank test and anxiety-depression scores by McNemar's test, comparing the original pre-treatment scores with those recorded 2 months after completion of therapy.

**Results**

*Anti-tumour effects*

The results of treatment are shown in Table I. At 75 and 100 mg m$^{-2}$, 5/8 patients (62%) with metastic breast cancer achieved an objective regression, one being complete (12%). At 125 mg m$^{-2}$ and 150 mg m$^{-2}$ 7/7 patients with metastatic breast cancer responded, four of them (57%) achieving complete remission. The patient with ovarian carcinoma present in each of the latter two groups also achieved a partial response, and so did 2/4 breast cancer patients in the control group (not treated with G-CSF). There were comparatively more complete responders in the two higher doses group ($P$=0.086), but this was a pilot study and the numbers were necessarily small. Nevertheless, an overall response rate of 80% (12/15) in patients with advanced breast cancer treated with G-CSF and a median time to progression of 6 months, range 2–9 months (Figure 2), are encouraging. Two patients, numbers 3 and 6, died from progressive disease within 6 weeks of therapy. Both had a very poor pre-treatment performance score (WHO grade 3) and failed to achieve significant regression of tumour burden. Similarly, one patient (number 19) in the control group, not given G-CSF, died after one course of chemotherapy from progressive disease. Tumour responses were

**Figure 2** Time to progression (in months) for the 14 patients treated with G-CSF who achieved a partial or complete response to chemotherapy. Disease recurrence in the complete responders was documented by biopsy.

**Figure 3** **a**, Median absolute neutrophil counts mm$^{-3}$ (ANC) of patients receiving 75 mg m$^{-2}$ of doxorubicin with (○) and without (△) G-CSF (four patients each) and 100 mg m$^{-2}$ with G–CSF (□, five patients). The heavy line denotes the peripheral neutrophil profile of the control group. Shaded areas represent the total area of neutropenia (ANC <1,000 mm$^{-3}$) following the first doxorubicin dose at 75 mg m$^{-2}$. **b**, Median ANC of patients receiving G-CSF and doxorubicin at 125 mg m$^{-2}$ (○, six patients) and 150 mg m$^{-2}$ (□, two patients).

also documented by measuring serum levels of tumour markers. Three of five patients who achieved a complete response had significantly raised pre-treatment levels of MCA, which in each case returned to normal within 2 months. A similar fall was also found in the CA15-3 levels of these three patients. Following disease progression/relapse patients were offered further therapy with cyclophosphamide, methotrexate and 5-fluorouracil. Seven patients are now assessable for response to second line chemotherapy and three have obtained objective responses (42%).

*Haematological changes*

Total and differential white counts were measured three times per week during the study. The absolute neutrophil counts (ANC) are shown in Figure 3. The ANC rose to normal or above normal levels by day 12–14 at all dose levels of doxorubicin given with G-CSF infusions, whereas an ANC > $2.5 \times 10^9 l^{-1}$ was not reached until day 19–21 after $75 mg m^{-2}$ of doxorubicin given without G-CSF. G-CSF infusion was followed by a rapid increase in peripheral neutrophil counts up to $40 \times 10^9 l^{-1}$. The nadir was usually on day 7 post-chemotherapy when G-CSF was given, but it occurred on days 12–13 when G-CSF was not given. The nadir and duration of neutropenia were increased for the two higher dose levels of doxorubicin (Figure 3b) compared with the two lower ones (Figure 3a). At $125 mg m^{-2}$ recovery from neutropenia following the third cycle of chemotherapy was slower than following the first two cycles. Platelet transfusions were required by 4/8 patients at the two higher dose levels, but no bleeding complications were seen. Blood transfusions were required by 4/9 patients in the two lower doses group and by 7/8 in the other, but in no case did the haemoglobin drop below $8 g dl^{-1}$. There were no life-threatening infections, but 7/8 patients receiving 125 and $150 mg m^{-2}$ required intravenous antibiotics at least once because of pyrexia and mucositis. Only one pyrexial episode was associated with positive blood cultures.

*Non-haematological toxicities*

Overall toxicities are represented in Figure 4 as the percentage of patients experiencing significant side-effects by the end of the full treatment period. There was a marked difference between the group of patients at the lower dose levels of doxorubicin (Figure 4a) and the rest (Figure 4b). The former were managed as outpatients with the exception of overnight admissions for a blood transfusion at the completion of chemotherapy in four and one admission for an infective episode in two (Figure 4a). In contrast, 7/8 patients given the two higher dose levels (125 and $150 mg m^{-2}$) had to be admitted. All patients developed mucositis resulting in three patients requiring parenteral nutrition, and in two cases this toxicity caused a 1 week delay in the final cycle of chemotherapy. The difference in the severity of mucositis between the two groups was statistically significant ($P = 0.002$). New epithelial toxicities were also found. The most dramatic one was the development of erythema of palms and soles by 7/8 patients in the higher dose group, which in some cases progressed to superficial blistering and desquamation. Again, the difference between the two groups was statistically significant ($P < 0.005$). A milder epithelial reaction also involved the vulva and perineum of 3/8 patients associated with vaginal candidiasis in two. Diarrhoea was reported by some patients but it was always tolerable and easily controlled. All these epithelial toxicities cleared within 2 weeks.

No patient has developed clinical cardiotoxicity. The mean resting LVEF for all patients was 44% (range 40–60%) before therapy, 48% after receiving $225 mg m^{-2}$, 45% after receiving $300 mg m^{-2}$ and 39% at $375 mg m^{-2}$. Two patients at the latter cumulative dose dropped their LVEF by 15%, but none have developed clinical signs of cardiac failure.

*Quality of life*

Fourteen patients receiving G-CSF completed quality of life questionnaires before, immediately after chemotherapy and, again, after a further 2 months. Eight scored above the threshold of the psychological subscale before treatment, and in five of these the scores increased at the end of chemotherapy, indicating worsening of depression and/or anxiety. However, all but one patient recorded scores within the normal range 2 months after the completion of treatment ($P < 0.05$). Thirty-five per cent of patients had normal functional status before therapy. Of the remaining 65%, functional status improved significantly in 42%, did not change in 15% and deteriorated in 8% ($P < 0.05$). Twelve of 14 patients reported pain before therapy and 10 of these patients had improved by 2 months after it. Similarly, 5/7 patients who had complained of breathlessness in the original questionnaire reported an improvement in the last one.

*Doxorubicin pharmacokinetics*

There was a linear increase in the area under the time–drug concentration curve (AUC) over the full dose range used. The kinetic parameters obtained suggest that there is no change in drug distribution with increased dose (Bronchud *et al.*, 1988).

**Figure 4** Toxicities (WHO coded) represented as percentage of patients developing cytotoxic-related side-effects during and after chemotherapy. See text for explanation. **a**, Doxorubicin 75 or $100 mg m^{-2}$ every 2 weeks: 1, alopecia (WHO 2, 3); 2, nausea/vomiting (WHO 2); 3, mucositis (WHO 2); 4, mucositis (WHO 3); 5, mucositis (WHO 4); 6, diarrhoea (WHO 2); 7, i.v. antibiotics (WHO 2, 3); 8, platelet transfusions (no bleeding); 9, red hands/feet (WHO 1); 10, vulvitis (WHO 2); 11, cardiotoxicity (WHO 1). **b**, Doxorubicin 125 or $150 mg m^{-2}$ every 2 weeks: 1, alopecia (WHO 2, 3); 2, nausea/vomiting (WHO 2); 3, mucositis (WHO 2); 4, mucositis (WHO 3); 5, mucositis (WHO 4); 6, diarrhoea (WHO 2); 7, i.v. antibiotics (WHO 2, 3); 8, platelet transfusions (no bleeding); 9, red hands/feet (WHO 2); 10, red hands/feet (WHO 3); 11, vulvitis (WHO 2); 12, cardiotoxicity (WHO 1); 13, cardiotoxicity (WHO 2).

### Discussion

Our previous study in patients with small cell lung cancer was the first to show that neutropenia and infection could be reduced by G-CSF following intermittent 'conventional' 3-weekly chemotherapy (Bronchud *et al.*, 1987). The study reported here shows that infusions of G-CSF allow dose escalations of chemotherapy and a decrease in the interval between doses. For example, if we take $300 mg m^{-2}$ as the total dose administered, it would take a patient receiving $75 mg m^{-2}$ every 3 weeks a minimum of 9 weeks to complete therapy. This total dose can be administered in 4 weeks ($100 mg m^{-2}$ every 2 weeks) or in 2 weeks ($150 mg m^{-2}$) under G-CSF cover. This represents an increase in dose intensity of 2.2–4.5-fold. No more than $375 mg m^{-2}$ total dose could be given at this dose intensity because of dose-limiting toxicities: severe mucositis and a severe desquamative skin rash in areas with high epithelial turnover rate. In addition, some non-clinical cardiotoxicity was also seen at this cumulative dose. This distinct difference in toxicity between the two lower and the two higher doses of chemotherapy, particularly after the second and third cycles, made us consider whether the kinetics of doxorubicin were non-linear. However, examination of the pharmacokinetic profiles indicated a linear relationship between dose and AUC for the range of doses employed and intracellular accumulation of doxorubicin has been suggested as responsible for the increase in toxicities (Bronchud *et al.*, submitted). None of the toxicities encountered were thought to be related to G-CSF therapy.

An overall response rate of 80% in patients with advanced breast cancer, with a median time to progression of 6 months, was achieved in this preliminary phase II study. Response rates were probably higher at the higher doses, but the numbers were too small to reach statistical significance. Two months after doxorubicin-G-CSF therapy there was a pronounced improvement of symptoms compared with before treatment. These results are comparable, if not better, to those obtained with high dose chemotherapy and bone marrow transplantation (Antman & Gale, 1988; Hortobagyi, 1988), which is only available in a few specialised centres and carries a significant mortality (10–20%) and morbidity. In addition, bone marrow rescue is difficult to repeat and may not be appropriate in those with marrow involved with malignancy or in those without a suitable related donor.

The importance of dose intensity has previously been reported for a variety of malignancies (Frei & Canellos, 1980; Cortes *et al.*, 1978; Jones *et al.*, 1987; De Vita, 1986; De Vita *et al.*, 1987). Many years ago Skipper showed that in experimental tumour models high dose intensity may make the difference between 50–100% cures and no cures at all, with equitoxic cytotoxic regimens (Skipper, 1967). However, most of the clinical data on the impact of dose–response in breast cancer come from retrospective studies, and there is a need for more clinical trials to assess the importance of both dose intensity and total dose in solid tumours (Henderson *et al.*, 1988). The availability of G-CSF will now allow direct testing of more intensive chemotherapy regimens in a variety of human cancers. The use of combination chemotherapy should further improve the clinical results of this new approach. It may give considerable benefits to patients with advanced cancer and possibly increase cure rates when used as an adjuvant to surgery.

We thank the Cancer Research Campaign and the Leukaemia Research Fund for their support. We would also like to thank J. Margison and J. Turnbull for technical assistance, R. Swindell for the statistical analysis of data, the Isotope Department and the Day Ward nursing staff at the Christie Hospital for their cooperation. We also wish to express our appreciation to M. Wyre for help in managing the implementation of the clinical protocol.

### References

ANTMAN, K. & GALE, R.P. (1988). Advanced breast cancer: high-dose chemotherapy and bone marrow autotransplants. *Ann. Intern. Med.*, **108**, 570.

BRINKLEY, D. (1985). Quality of life in cancer trials. *Br. Med. J.*, **291**, 685.

BRONCHUD, M.H., POTTER, M.R., MORGENSTERN, G. and 8 others (1988). *In vitro* and *in vivo* analysis of the effects of recombinant human granulocyte colony-stimulating factor in patients. *Br. J. Cancer*, **58**, 64.

BRONCHUD, M.H., SCARFFE, J.H., THATCHER, N. and 5 others (1987). Phase I/II study of recombinant human granulocyte colony-stimulating factor in patients receiving intensive chemotherapy for small cell lung cancer. *Br. J. Cancer*, **56**, 809.

CARMO-PEREIRA, J., OLIVEIRA COSTA, F., HENRIQUES, E. and 4 others (1987). A comparison of two doses of adriamycin in the pirmary chemotherapy of disseminated breast carcinoma. *Br. J. Cancer*, **56**, 471.

CORTES, E.P., HOLLAND, J.F. & GLIDEWELL, O. (1978). Amputation and adriamycin in primary osteosarcoma: a five year report. *Cancer Treat. Rep.*, **62**, 271.

DE HAES, J.C.J.M., PRUYN, J.F.A. & VAN KNIPPENBERG, F.C.F. (1983). Klachtenlijst voor Kankerpatienten. Eerste ervaringen. *Nederlands Tijdsch. Psychol.*, **38**, 403.

DE VITA, V.T. (1986). Dose–response is alive and well. *J. Clin. Oncol.*, **4**, 1157.

DE VITA, V.T. HUBBARD, S.M. & LONGO, D.L. (1987). The chemotherapy of lymphomas: looking back, moving forward. The Richard and Hinda Rosenthal Foundation Award Lecture. *Cancer Res.*, **47**, 5810.

FREI, E. & CANELLOS, G.P. (1980). Dose: a critical factor in cancer chemotherapy. *Am. J. Med.*, **69**, 585.

HAYWARD, J.L., RUBENS, R.D., CARBONE, P.P., HENSON, J.C., KUMAOKA, S. & SEGALOFF, A. (1977). Assessment of response to therapy in advanced breast cancer. *Br. J. Cancer*, **35**, 292.

HENDERSON, I.C., HAYES, D.F. & GELMAN, R. (1988). Dose–response in the treatment of breast cancer. *J. Clin. Oncol.*, **6**, 1501.

HOOGSTRATEN, B. (1975). Adriamycin (NSC-123127) in the treatment of advanced breast cancer: studies by the Southwest Oncology Group. *Cancer Chemother. Rep.*, **6**, 329.

HORTOBAGYI, G.N. (1988). The role of high-dose chemotherapy with autologous bone marrow transplantation in the treatment of breast cancer. *Bone Marrow Transplan.*, **3**, 525.

HUBBARD, S.M., BARKES, P. & YOUNG, R.C. (1978). Adriamycin therapy for advanced ovarian carcinoma recurrent after chemotherapy. *Cancer Treat. Rep.*, **62**, 1375.

ISRAEL, M., PEGG, W.J., WILKINSON, P.M. & GARNICK, M.B. (1978). Liquid chromatographic analysis of adriamycin and metabolites in biological fluids. *J. Liquid Chromatogr.*, **1**, 795.

JONES, R.B., HOLLAND, J.F., BHARDWAJ, S., NORTON, L., WILFINGER, C. & STRASHUN, A. (1987). A phase I-II study of intensive dose adriamycin for advanced breast cancer. *J. Clin. Oncol.*, **5**, 172.

SKIPPER, H.E. (1967). Criteria associated with destruction of leukemia and solid tumor cells in animals. *Cancer Res.*, **27**, 2636.

SOUZA, L.M., BOONE, T.C., GABRILOVE, J. and 11 others (1986). Recombinant human granulocyte colony-stimulating factor: effects on normal and leukemic myeloid cells. *Science*, **232**, 261.

STEINER, R., STEWART, J.F., CANTWELL, B.M.J., MINTON, M.J., KNIGHT, R.K. & RUBENS, R.D. (1983). Adriamycin alone or combined with vincristine in the treatment of advanced breast cancer. *Eur. J. Cancer Clin. Oncol.*, **19**, 1553.

WHEELER, R.H., ENSMINGER, W.D., THRALL, J.H. & ANDERSON, J.L. (1982). High-dose doxorubicin: an exploration of the dose–response curve in human neoplasia. *Cancer Treat. Rep.*, **66**, 493.